# ARIMA and ARIMA-ERNN models for prediction of pertussis incidence in mainland China from 2004 to 2021

**DOI:** 10.1186/s12889-022-13872-9

**Published:** 2022-07-29

**Authors:** Meng Wang, Jinhua Pan, Xinghui Li, Mengying Li, Zhixi Liu, Qi Zhao, Linyun Luo, Haiping Chen, Sirui Chen, Feng Jiang, Liping Zhang, Weibing Wang, Ying Wang

**Affiliations:** 1 School of Public Health, Fudan University, Shanghai, 200032 China; 2 NHC Key Laboratory of Health Technology Assessment, Fudan University, Shanghai, 200032 China; 3 Department of Ultrasound Medicine, The First Affiliated Hospital, School of Medicine, Zhejiang University, Hangzhou, 310003 China; 4 Key Laboratory of Pulsed Power Translational Medicine of Zhejiang Province, Zhejiang University, Hangzhou, 310003 China; 5 Key Laboratory of Public Health Safety of Ministry of Education, Fudan University, Shanghai, 200032 China; 6 China National Biotec Group Company Limited, Beijing, 100024 China; 7 Hunan Normal University, Hunan, 410081 China; 8 Institute of Expanded Programme On Immunization, Guizhou Provincial Center for Disease Control and Prevention, Guizhou Province, Guiyang, 550004 People’s Republic of China; 9 Minhang Center for Disease Control and Prevention, Shanghai, 201100 China

**Keywords:** Pertussis, ARIMA model, ARIMA-ERNN model, Predictive effect

## Abstract

**Objective:**

To compare an autoregressive integrated moving average (ARIMA) model with a model that combines ARIMA with the Elman recurrent neural network (ARIMA-ERNN) in predicting the incidence of pertussis in mainland China.

**Background:**

The incidence of pertussis has increased rapidly in mainland China since 2016, making the disease an increasing public health threat. There is a pressing need for models capable of accurately predicting the incidence of pertussis in order to guide prevention and control measures. We developed and compared two models for predicting pertussis incidence in mainland China.

**Methods:**

Data on the incidence of pertussis in mainland China from 2004 to 2019 were obtained from the official website of the Chinese Center for Disease Control and Prevention. An ARIMA model was established using SAS (ver. 9.4) software and an ARIMA-ERNN model was established using MATLAB (ver. R2019a) software. The performances of these models were compared.

**Results:**

From 2004 to 2019, there were 104,837 reported cases of pertussis in mainland China, with an increasing incidence over time. The incidence of pertussis showed obvious seasonal characteristics, with the peak lasting from March to September every year. Compared with the mean squared error (MSE), mean absolute error (MAE), and mean absolute percentage error (MAPE) of the ARIMA model, those of the ARIMA-ERNN model were 81.43%, 95.97% and 80.86% lower, respectively, in fitting performance. In terms of prediction performance, the MAE, MSE and MAPE were 37.75%, 56.88% and 43.75% lower, respectively.

**Conclusion:**

The fitting and prediction performances of the ARIMA-ERNN model were better than those of the ARIMA model. This provides theoretical support for the prediction of infectious diseases and should be beneficial to public health decision making.

## Introduction

Pertussis (whooping cough) is an acute and highly contagious pulmonary disease caused by a small aerobic Gram-negative bacterium, *Bordetella pertussis *[1]. Pertussis can occur in adults and children, but is often more serious in children, particularly very young infants. Worldwide, pertussis is one of the top ten causes of death during childhood [2]. A 2012 study of pertussis estimated that there were about 30 to 50 million cases and 300,000 deaths per year globally [3], and a 2014 study estimated that there were 24.1 million cases and 160,700 deaths per year globally in children younger than 5 years [4]. In 2018, the WHO estimated that there were approximately 150,000 cases of pertussis worldwide [5]. However, pertussis is often overlooked or misdiagnosed because in many patients it presents with only mild clinical symptoms [6], leading to a possible underestimation of its morbidity [7]. Recent studies of the epidemiology of pertussis reported an epidemic cycle, with increasing numbers of patients every 3 years (on average) [8] in countries such as Canada, Australia, and China [9, 10]. Several other recent studies reported that the incidence of pertussis in China has risen sharply during recent years [11, 12]. In China, for example, the median total economic burden for each case of pertussis in 2017 and 2018 was 8603 Yuan in Yantai (Shangdon) [13], and the average direct economic burden of each inpatient with pertussis in 2019 was 13,291 Yuan in Chongqing[14]. Thus, the resurgence of pertussis is a major financial and public health problem in China.

It is necessary to forecast changes in the morbidity of pertussis so that effective strategies can be implemented for prevention and control, and so that associated health hazards and economic losses can be reduced. There are currently two general types of time series forecasting models that are widely used in epidemiological forecasting. Conventional time series analysis models construct a model using historical data and mainly rely on the linear features of the data; these include the Grey model, Markov model, and autoregressive integrated moving average (ARIMA). A time series may also be analyzed using machine learning theory, in which a model is constructed using an artificial neural network (ANN) to capture the nonlinear features of the data. ARIMA models are the best-known model for time series forecasting, and have been used by many researchers to predict infectious diseases that have characteristic seasonal outbreaks [15]. However, an ARIMA model does not consider nonlinearities in a time series [16].

Given the shortcomings of ARIMA models, there is increasing interest in using ANN models for epidemiological time series forecasting [17] because these models account for nonlinearities in the data. Most of the ANN models used in epidemiological forecasting are based on feed-forward ANNs (static neural networks), such as the back-propagation neural network and the generalized regression neural network. Due to the aggregation and variation of infectious diseases, feed-forward ANNs may not be suitable for analyzing epidemiological data [18]. Unlike feed-forward neural networks, the Elman recurrent neural network (ERNN) can model dynamic information because it uses of additional memory neurons and local feedback [3]. The ability of the ERNN to model dynamic information and its strong sensitivity to time series data thus make it suitable for modeling infectious diseases. Although ANNs can successfully model nonlinear data, they often fail to capture the linear features of the data. Real world time series often contain linear and nonlinear components [19] hence, a model should capture both of these patterns [20]. Therefore, the combined use of an ARIMA model and an ERNN model may provide superior performance [21].

A wide range of epidemiological research has been conducted on pertussis, with most studies focusing on factors that influenced its incidence [22–26]. Very few reports have focused on predicting the incidence of pertussis. Two recent studies used ARIMA to predict the incidence of pertussis. Raycheva R et al. [27] developed an ARIMA (3, 0, 0) model that adequately reflected trends in pertussis incidence and predicted recent disease dynamics with acceptably low errors. Zeng et al. [12] used ARIMA to analyze pertussis data from January 2005 to June 2016 in China; they found that an ARIMA(0,1,0)(1,1,1)12 model showed the best performance. Another study used a seasonal ARIMA model combined with a nonlinear autoregressive network (SARIMA-NAR) model to forecast the incidence of pertussis in China, and found that using this combination of models greatly improved the accuracy of predictions [11]. In this research, we compared the abilities of an ARIMA-ERNN model and an ARIMA model to predict incidence of pertussis in China. We evaluated the performance of these models by calculating the mean squared error (MSE), mean absolute error (MAE), and mean absolute percentage error (MAPE).

## Materials and Methods

### Data sources

Monthly data on all cases of pertussis from January 2004 to December 2019 in mainland China (excluding Hong Kong, Macao Special Administrative Region, and Taiwan) were obtained from the official website of the Chinese Center for Disease Control and Prevention (China CDC, http://www.chinacdc.cn/). Annual data on cases during the same period were obtained from the National Bureau of Statistics of China (http://www.stats.gov.cn/tjsj/ndsj/). Pertussis is classified as a Class B notifiable disease in China, and has been reported through China's National Disease Report System (NDRS) network since 2004. Detailed criteria for the diagnosis of pertussis (WS 274–2007) were issued by the Chinese Ministry of Health on April 17, 2007 [28].

### Seasonal-trend decomposition using loess (STL)

STL can decompose a time series with seasonal characteristics into a long-term trend, a seasonal trend, and random effects. Thus, this method was used to analyze the seasonal characteristics and incidence of pertussis. Based on the monthly incidence rate of pertussis from 2004 to 2019, the original sequence was decomposed into three parts: a long-term trend, a seasonal trend, and a remainder. The STL plot was used to initially identify seasons that had a high incidence of pertussis.

### ARIMA Model

Box and Jenkins proposed the ARIMA model as a method for time series analysis and prediction. The basic idea of an ARIMA model is that it treats a data series formed by predicted objects over time as a random sequence. The relationship between these random sequences reflects the extensibility of the development of the predicted objects. This relationship is expressed by mathematical models and used for prediction. Generally, an ARIMA model can be classified as a simple ARIMA (p, d, q) model, a seasonal ARIMA (P, D, Q) S model, and a seasonal-product ARIMA (p, d, q) (P, D, Q) S model, where p, d, q and P, D, Q are the orders of the continuous and seasonal autoregressive terms, difference terms, and moving average terms, respectively. The essence of this model is that it extracts nonstationary deterministic information from a time series by calculating differences. When the residual sequence of an ARIMA model is random (white noise), the model is considered the best linear prediction model for short-term predictions of a time series.

### Elman Recurrent Neural Network

The Elman Recurrent Neural Network (ERNN) is a feedback-like (dynamic) neural network proposed by Jeffrey L. Elman and revised by Pham et al. It is a classical nonlinear local recursive network, which consists of an input layer, a hidden layer, a receiving layer, and an output layer. The receiving layer stores the output state of feedback using the delay operator to provide dynamic memorization, so that the system has timely reactions and accurately reflects the dynamics of a system. The self-connection mode of the hidden layer is more sensitive to the time series data. The internal feedback of the ERNN provides dynamic processing of data, and ignores the influence of external noise on the prediction model, thus enabling the model to map nonlinearities with high accuracy.

During the learning process of the ERNN, the dynamics between the input and output parameters are acquired from training data, and stable network parameters are then determined. The ERNN learning algorithm uses rules for error correction. First, input training data is processed through the input layer and the hidden layer, and the input signal is then propagated forward by the output results of the output layer. Then, the error between the predicted and measured values of the output layer is calculated, and if this error exceeds a pre-set threshold, it enters the error back-propagation. The error signals are propagated back to each layer of neurons by a certain form, layer by layer, and the connection weights and threshold matrices of neurons in each layer are updated and modified accordingly.

### ARIMA-ERNN Model

First, an optimal ARIMA model was constructed, and information extracted from the original sequence was used to construct an ANN. Second, the predicted values of the ARIMA model and the normalized data of the corresponding time series were used as input data and the normalized real values as the output data to establish an ERNN model that had two-dimensional input and one-dimensional output. Third, the ERNN model used the MSE of the error sequence to evaluate network performance using the continuous learning and training input data and output data. When the MSE was smallest, the ERNN was considered to have the best fit. Fourth, an inverse transformation was performed from the predicted value to establish the combined model. The error of the prediction model was reduced by nonlinear mapping of the ANN, and the advantages of the two models were thus synthesized to improve the prediction accuracy.

### Indicators of model performance

The statistical fits and accuracies of prediction of the selected models were measured using three metrics, MSE, MAE, and MAPE, in which smaller values indicated a better model [11, 29].

1 $$MSE=\frac{1}{N}\sum_{i=1}^{N}{({X}_{i}-{\overline{X} }_{i})}^{2}$$

2 $$MAE=\frac{1}{N}\sum_{i=1}^{N}\left|{X}_{i}-{\overline{X} }_{i}\right|$$

3 $$MAPE=\frac{1}{N}\sum_{i=1}^{N}\frac{\left|{X}_{i}-{\overline{X} }_{i}\right|}{{X}_{i}}$$

where ${X}_{i}$ is the actual value at time i, ${\overline{X} }_{i}$ is the predicted value at time i, and N is the number of cases.

### Data analysis

Microsoft Excel (2016) was used for data collation and statistical descriptions, and R software (Version 3.6.0) was used for plotting seasonal breakdowns, monthly changes, and time series. The ARIMA model was developed using SAS version 9.4, and the ARIMA-ERNN model was developed using MATLAB version R2019a.

## Results

### Time Distribution of Pertussis

#### Changes in Pertussis Incidence

From 2004 to 2019, 104,837 cases of pertussis were reported in mainland China, with an increasing incidence over time (Table 1). Compared with 2004 (4705 cases), the incidence of pertussis was 538% greater in 2019 (30,027 cases).

**Table 1:** Incidence of pertussis in mainland China from 2004 to 2019

Year	Population(100 thousand)	Reported cases(cases)	Incidence Rate(per 100,000)
2004	12,997.23757	4705	0.362
2005	12,998.79873	3844	0.2957
2006	13,074.94867	2547	0.1948
2007	13,143.24818	2881	0.2192
2008	13,209.73990	2387	0.1807
2009	13,278.41845	1612	0.1214
2010	13,343.41906	1764	0.1322
2011	13,409.69632	2517	0.1877
2012	13,475.30864	2183	0.162
2013	13,544.30380	1712	0.1264
2014	13,550.69583	3408	0.2515
2015	13,623.90014	6658	0.4887
2016	13,706.43103	5584	0.4074
2017	13,900.80000	10,542	0.7584
2018	13,953.80000	22,466	1.6100
2019	14,000.50000	30,027	2.1501
total	13,450.7	104,837	0.4780

### Seasonal Pattern of Pertussis

**Fig. 1:**
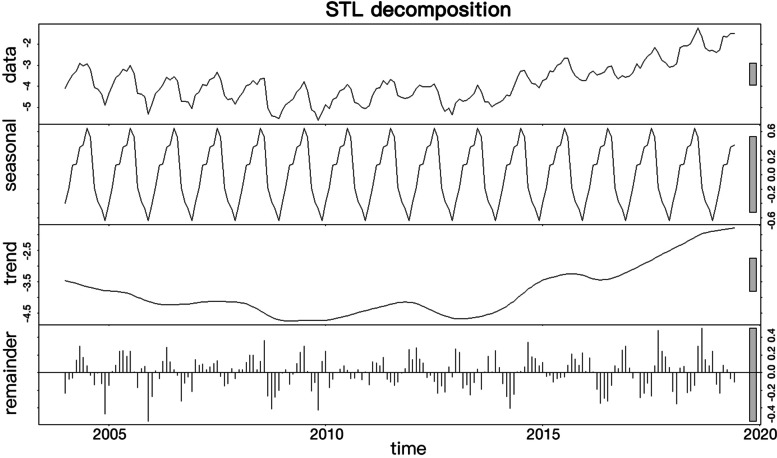
Seasonal decomposition (STL) of the incidence of pertussis from January 2004 to June 2019

Analysis of the raw data indicated that the incidence of pertussis had a seasonal pattern with a period of 1 year (Fig. 1, top). Further analysis of these data using STL indicated an obvious seasonal pattern with a long-term trend indicating declining incidence, followed increasing incidence (Fig. 1, middle). The STL method provided a reliable extraction of seasonal information and trend, as indicated by the remainder plot, which showed that the errors were evenly distributed (Fig. 1, bottom).

**Fig. 2:**
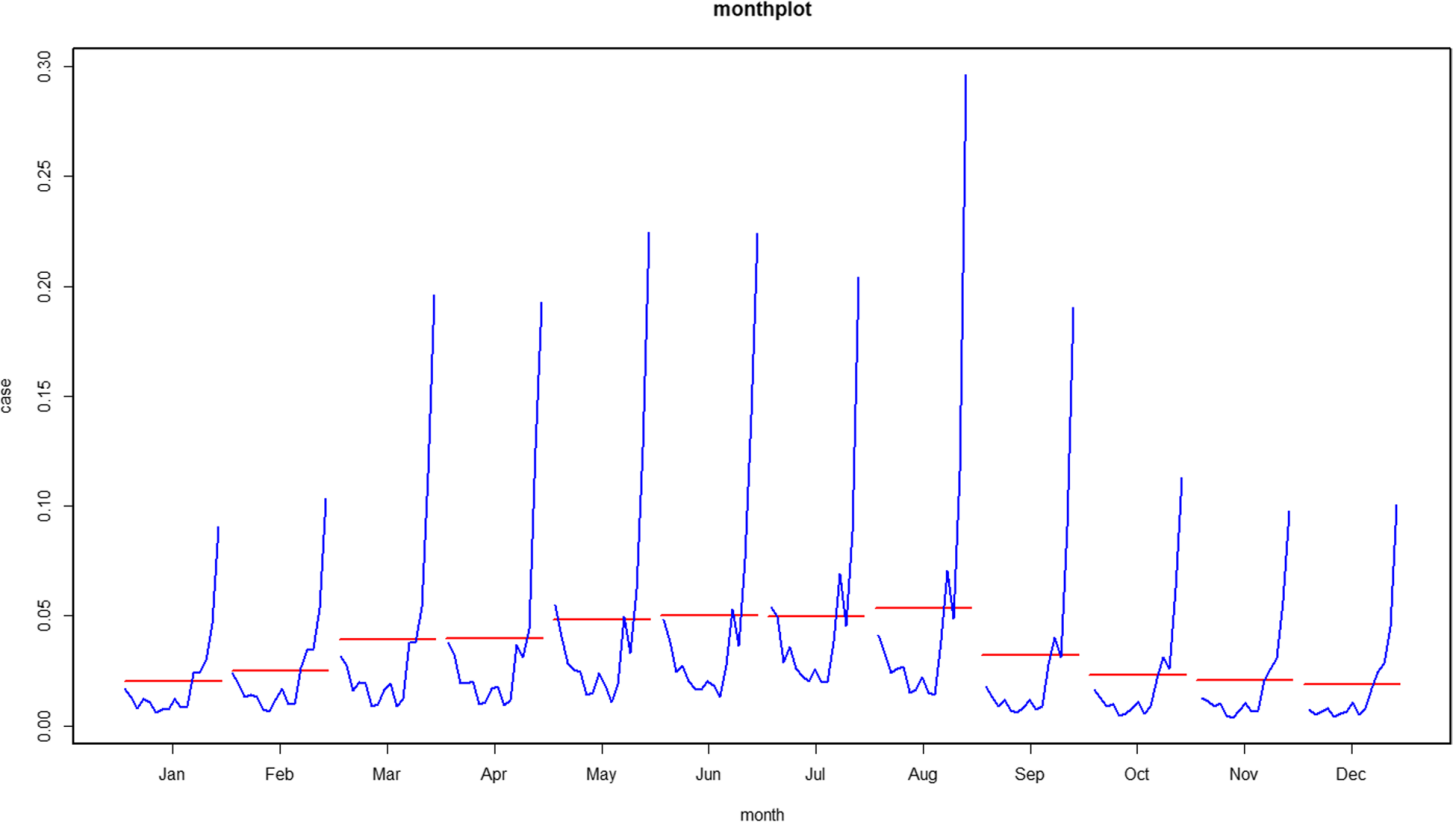
Pertussis incidence rates during each month from 2004 to June 2019

The STL results can only approximate the seasonal characteristics and long-term trend of a disease, and cannot determine the peak season. Thus, we also examined these data as a “monthly plot”, which presents the changing incidence from 2004 to June 2019 during each month (Fig. 2). These results indicated that August had the most reported cases, and the period of March to September had high incidence rates.

### ARIMA Model

**Fig. 3:**
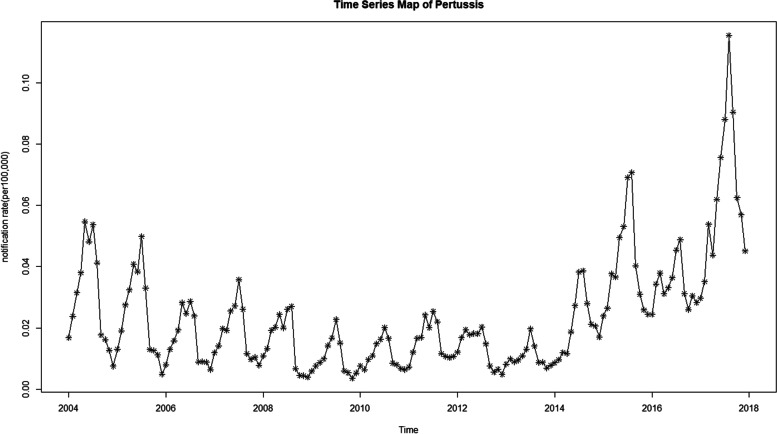
Monthly incidence of pertussis from 2004 to 2017

We developed the ARIMA model using the monthly incidence data of pertussis cases from January 2004 to December 2017 as a training set and the monthly incidence data from January 2018 to June 2019 as a validation set. The raw data indicated a slow decline, followed by a significant increase (Fig. 3).

The unit root test was used to determine the stationarity of the data. For an alpha level of 0.05, the results of this test showed that the original series was stationary after accounting for the first-order difference and seasonal difference (*P* < 0.05). We then established an ARIMA model for the adjusted sequence and examined the results using the white noise test (Table 2). These results showed that the adjusted sequence was not a white noise sequence, and that an ARIMA model could be established. Because the original series had a period of 12 months and became stable after accounting for the first-order difference and seasonal difference, we used an ARIMA (p, 1, q) (P, 1, Q) 12 model.

**Table 2:** White noise test of the adjusted sequence

To lag	Chi-Square	DF	Pr > ChiSq	Atocorrelations					
6	8.47	6	0.2055	0.053	0.087	0.036	-0.143	-0.127	-0.065
12	31.74	12	0.0015	-0.043	-0.007	0.136	-0.004	0.106	-0.325
18	41.52	18	0.0013	-0.078	-0.150	-0.132	-0.084	0.054	0.023
24	49.12	24	0.0018	0.069	0.022	-0.096	-0.002	-0.137	0.091

### ARIMA Model Recognition and Order Determination

**Fig. 4:**
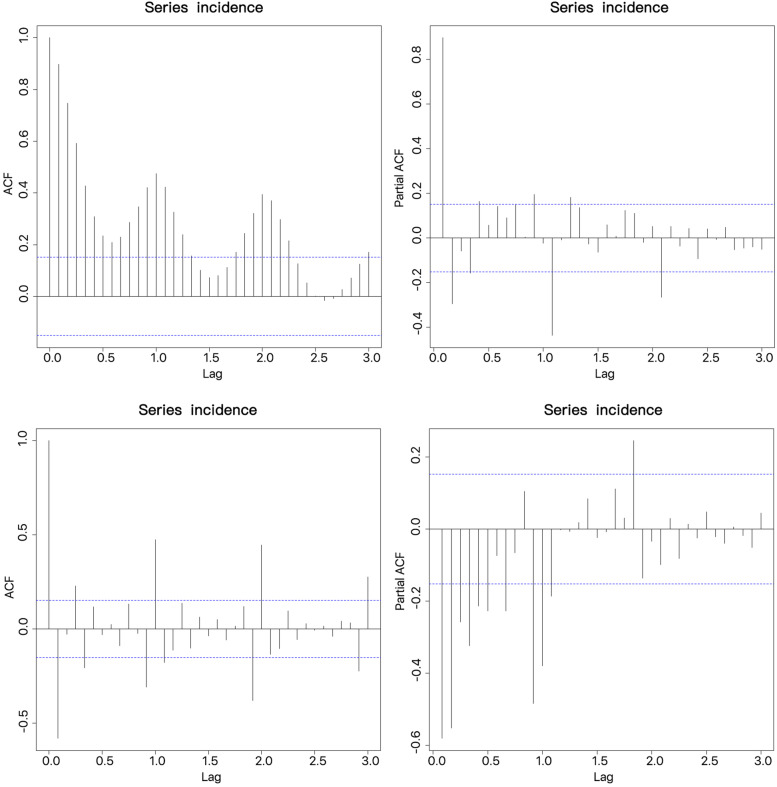
ACF and PACF of differenced pertussis incidence series. ACF, autocorrelation function; PACF, partial autocorrelation function

Next we performed model recognition procedures for the ARIMA model (Fig. 4). In particular, we applied the autocorrelation function (ACF) and partial autocorrelation function (PACF) of the adjusted sequence to determine the values of P, Q, p, and q. A white noise test of the residuals (Table 2) indicated that the information of the fitted model was extracted completely, and that the ARIMA model had parameters of (1,1,1) (0,1,1) 12.

### Model Validation

We then used an ARIMA (1,1,1) (0,1,1) 12 model to predict the incidence of pertussis in mainland China from January 2018 to June 2019. The MSE of this model was 0.00937, indicating high accuracy.

### ARIMA-ERNN model

To develop the ARIMA-ERNN model, we first used the fitted values and corresponding times of the seasonal product ARIMA (1,1,1) (0,1,1) 12 model to train the network.

### Sample Set Partition

Due to the establishment of an optimal ARIMA model for first-order and seasonal differences, the number of predicted values declined by 13 samples. In the second step, we used data from February 2005 to December 2017 as training data and an internal validation period of January 2017 to December 2017, and then tested the model using external validation for the period of January 2018 to December 2019. The input and output data were normalized, and then network training was carried out using the mapminmax function in MATLAB.

### Construction of the ARIMA-ERNN model

The following empirical formula was used to determine the number of neurons in the hidden layer (*N*):

$$N=\sqrt{n+m}+a$$

where m is the number of neurons in the input layer, n is the number of neurons in the output layer, and a is a constant [1, 10]. According to this calculation, the hidden layer of the ERNN had 3 to 12 neurons. We used a Tan-Sigmoid function for the implicit layer of ERNN, a Purelin function for the output layer, traingdx for the training function, Learngdm for the network weight learning function, and MSE to assess model performance. The parameters of the network were as follows: 10,000 iteration steps, learning rate of 0.01, and learning objective (learning error) of 0.004. We then used an ERNN with a structure of 2–9-1 structure to predict the incidence of pertussis. The MSE of the ARIMA-ERNN model was 0.00077, better than that of the ARIMA model (0.00937).

### Model Prediction

**Fig. 5:**
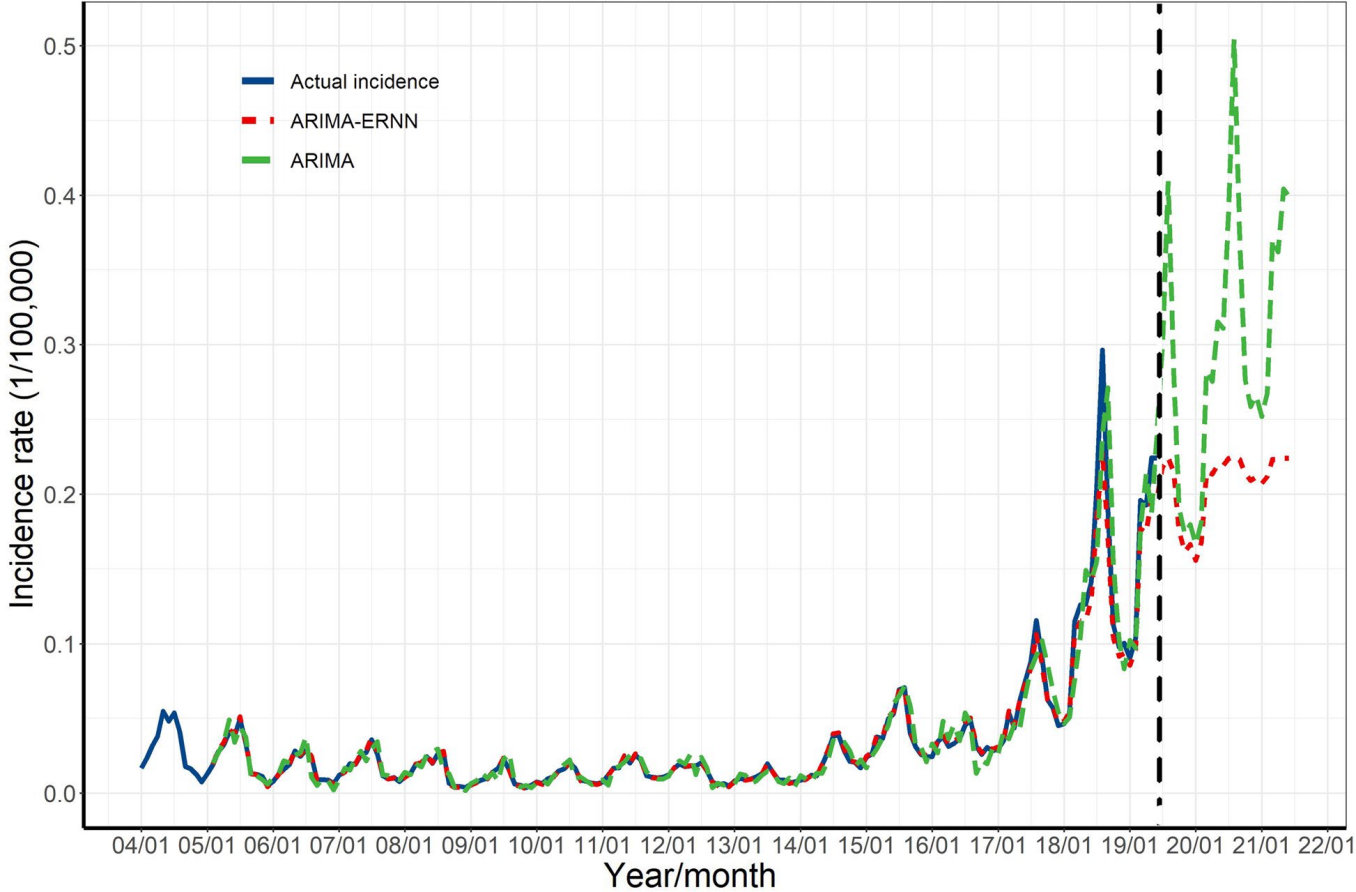
Predictions of the incidence of pertussis in China from the ARIMA model and the ARIMA-ERNN model. Statistical fits: left of the vertical dashed line; predictions: right of the vertical dashed line

Next we used the ARIMA model and the ARIMA-ERNN model to predict the incidence of pertussis in China from July 2019 to June 2021 (Fig. 5), and compared these models by calculating of MSE, MAE, and MAPE (Table 3). All three of these error values were lower for the ARIMA-ERNN model than for the ARIMA model, indicating that the ARIMA-ERNN model performed better.

**Table 3:** Comparison of the performance of the ARIMA and ARIMA-ERNN models

Model	Fitting performance			Prediction performance		
	MAE	MSE	MAPE (%)	MAE	MSE	MAPE (%)
ARIMA	0.004222	0.000037143	21.06%	0.024864	0.001069	15.68%
ARIMA-ERNN	0.000784	0.000001498	4.03%	0.015479	0.000461	8.82%

## Discussion

The introduction of the pertussis vaccine greatly reduced the threat of this disease. However, a resurgence of pertussis has occurred in many countries, including China, and pertussis remains a challenging public health problem in China and elsewhere. Therefore, the ability to accurately predict the incidence of pertussis would assist in the implementation of appropriate public health interventions. This study compared an ARIMA model with an ARIMA-ERNN model in predicting the incidence of pertussis in mainland China. We found that an ARIMA (1,1,1) (0,1,1) 12 model provided highly accurate predictions of the incidence of pertussis in mainland China from January 2018 to June 2019. This is not consistent with the best ARIMA used in the previous two studies [12, 27], presumably due to the use of data from different years.

In other fields, such as economics and transportation, the ARIMA-ERNN model has been found to provide better predictive accuracy than other models [30, 31]. However, epidemiologists have only rarely used the ARIMA-ERNN model for the prediction of infectious diseases [32]. To the best of our knowledge, the present study constitutes the first use of a combined ARIMA-ERNN model to predict the incidence of pertussis. Compared with the ARIMA model, the statistical fit of our ARIMA-ERNN model had an 81.43% lower MAE, 95.97% lower MSE, and 80.86% lower MAPE, and the model predictions had a 37.75% lower MAE, 56.88% lower MSE, and 43.75% lower MAPE. Thus, the statistical fit and predictions of the combined model were better than those of the single ARIMA model, consistent with previous researches [11, 29]. We attribute these findings to the superior ability of the ARIMA-ERNN model to capture the linear and nonlinear characteristics of the sequence, and to reduce the loss of information. At the same time, the ERNN contains a local topological recursive structure, which makes it more tolerant [20] and provides certain advantages in dynamic modeling compared with a static neural network [3, 33]. We believe that these characteristics of the ERNN give the ARIMA-ERNN model a better ability to characterize the dynamic information in the time series data.

Compared with the results of two other studies [11, 12], our ARIMA-ERNN model also provided better accuracy. The MAPE is the most commonly used measure of model accuracy due to its scale-independency and easy interpretability [34]. Analysis of the statistical fit indicated that the MAPE value of our ARIMA-ERNN model was 76.96% lower than reported for an ETS model and 52.59% lower than reported for a novel wavelet-based SARIMA-NAR hybrid model. This increased prediction accuracy may be due to our use of more monthly data. Specifically, we used 18 months as the forecast set, whereas previous studies [11, 12] used only 6 months as the forecast set. We also calculated the MAPE of the forecast set from January to June 2018 to ensure the accuracy of comparison. Our MAPE was 6.53%, slightly lower than reported in the previous study (6.70%), confirming that our model was more accurate. Thus, our research indicated that the ARIMA-ERNN model was highly effective in predicting the incidence of pertussis, suggesting it may also have potential for predicting the incidence of similar infectious diseases.

The present research indicated that the incidence of pertussis in China did indeed increase, especially during 2018. This is consistent with previous research findings in China [11, 12, 35]. From 2004 to 2013, the incidence of pertussis in China had an overall downward trend. However, after 2014, there was a huge increase up to a rate of 2.15 per 100,000 in 2019, providing an important reminder that pertussis remains a threat in China. Similar to countries such as Canada, the United States, and Australia [36], the recurrence of pertussis has become an increasing problem in China. Previous studies indicated that the appearance of erythromycin-resistant B. pertussis and the evolution of B. pertussis might be the responsible for the increasing incidence in China [35]. In 2013, China completed the switch from the whole-cell pertussis vaccine (DTwP) to the diphtheria tetanus pertussis (DTaP) vaccine. Since 2013, three anti-PT IgG antibody detection kits have been approved in China, and nucleic acid PCR detection reagents were approved in 2019 [37]. We speculate that the change of vaccine type and the increased use of diagnostic testing may have contributed to the increased identification of cases, as in some developed countries [38]. In addition, unlike some developed countries, China does not implement a “cocooning strategy” [39, 40] for immunization and it does not have separate pertussis vaccines for adolescents and adults; these, two factors may also have contributed to the increase in incidence of pertussis. In general, we believe that the resurgence of pertussis cannot be attributed to any single factor, and that further studies are needed to determine the potential reasons for the increasing incidence in China.

We found a significant seasonality in the incidence of pertussis, with the greatest incidence during March to September. This result is consistent with several other studies [11, 12], but the nature of the seasonality of pertussis differs in different regions. For example, Leong et al. reported a peak incidence in Australia during spring and summer (November to January) [41], Guimarães et al. reported a peak incidence in Brazil during spring and autumn [42] and Hitz et al. reported a peak incidence in Germany during summer. Unfortunately, the reasons for the seasonality of pertussis remain mostly unknown. Some seasons may provide a more optimal environment for the pathogen, and the human immune response may also vary with the seasons. Thus, further studies are needed to examine the distribution and survival of *B. pertussis* and the mechanisms of underlying pathogenic factors [43].

This study had some limitations. All of our primary data were from a national database. Although China classifies pertussis as a Class B statutory infectious disease, the actual incidence of the disease is probably underestimated. Our research predicted the incidence rate for China overall, although there are likely to be large differences in incidence within China due to its large area and many regional differences. Moreover, we were unable to include some factors in our models that may affect the incidence of pertussis because the available data were not comprehensive. Future studies should seek to overcome these limitations.

## Conclusion

The present study compared predictions of pertussis incidence in mainland China obtained using an ARIMA model and an ARIMA-ERNN model. The results indicated that an ARIMA-ERNN model should be considered for monitoring the incidence of pertussis in China.

## Data Availability

The datasets generated and/or analyzed during the current study are available in the [official website of Chinese Center for Disease Control and Prevention] repository (http://www.chinacdc.cn/) and [National Bureau of Statistics of China] (http://www.stats.gov.cn/tjsj/ndsj/).
